# Embracing sex-specific differences in engineered kidney models for enhanced biological understanding of kidney function

**DOI:** 10.1186/s13293-024-00662-8

**Published:** 2024-12-02

**Authors:** Charlotte Veser, Aurélie Carlier, Vanessa Dubois, Silvia M. Mihăilă, Sangita Swapnasrita

**Affiliations:** 1 Utrecht Institute for Pharmaceutical Sciences, Universiteitsweg 99, 3584 CG Utrecht, The Netherlands; 2 MERLN Institute for Technology-Inspired Regenerative Medicine, Universiteitssingel 40, 6229 ER Maastricht, The Netherlands; 3 Basic and Translational Endocrinology (BaTE), Department of Basic and Applied Medical Sciences, Ghent University, Corneel Heymanslaan 10, 9000 Ghent, Belgium

**Keywords:** Sex-specific differences, Kidney function, In vitro models

## Abstract

In vitro models serve as indispensable tools for advancing our understanding of biological processes, elucidating disease mechanisms, and establishing screening platforms for drug discovery. Kidneys play an instrumental role in the transport and elimination of drugs and toxins. Nevertheless, despite the well-documented inter-individual variability in kidney function and the multifaceted nature of renal diseases—spanning from their origin, trigger and which segment of the kidney is affected—to presentation, progression and prognosis, few studies take into consideration the variable of sex. Notably, the inherent disparities between female and male biology warrants a more comprehensive representation within in vitro models of the kidney. The omission of sex as a fundamental biological variable carries the substantial risk of overlooking sex-specific mechanisms implicated in health and disease, along with potential differences in drug responsiveness and toxicity profiles between sexes. This review emphasizes the importance of incorporating cellular, biological and functional sex-specific features of renal activity in health and disease in in vitro models. For that, we thoroughly document renal sex-specific features and propose a strategic experimental framework to integrate sex-based differences into human kidney in vitro models by outlining critical design criteria to elucidate sex-based features at cellular and tissue levels. The goal is to enhance the accuracy of models to unravel renal mechanisms, and improve our understanding of their impact on drug efficacy and safety profiles, paving the way for a more comprehensive understanding of patient-specific treatment modalities.

## Abstract (plain language summary)

Understanding the differences in kidney function between males and females is crucial for developing safer and more effective drugs and drug dosing strategies. Sex differences arise from a combination of hormonal, anatomical, and genetic factors. However, many current kidney models used in research fail to accurately replicate these distinctions, hindering our understanding of kidney health, disease and drug effectiveness. To address this gap, this review delves into the existing knowledge about sex-related variations in kidney function and disorders. Additionally, it examines the limitations of current kidney models and suggests a framework for sex-inclusive kidney research. By doing so, the review provides critical insights and suggestions to develop improved models that enhance our understanding of kidney diseases and aid in drug discovery and safety assessments. Ultimately, this will lead to more efficient and secure drug development processes.

## Highlights

- Sex as a crucial variable: The review underscores the vital role of sex as a variable in kidney models, impacting current understanding of renal diseases and drug responses.
- Sexual dimorphism in kidney function: The review explores the origin of sexual dimorphism in kidney function through the lens of sex differences in kidney growth, transporter machinery, renal RAAS system, sex hormone receptors and inflammatory response, addressing clinical implications for nephrotoxicity screens, kidney diseases and hormone therapy.
- Proposal of a strategic framework: By highlighting a cohesive experimental framework, the review guides the development of more accurate kidney in vitro models, integrating sex-based distinctions for improved physiological and pathological studies.
- Implication for broader research: Beyond kidneys, the review advocates acknowledging and exploring sex-based disparities in the broader biomedical research field, crucial for a comprehensive understanding and the development of patient-specific treatments.

## Introduction

The kidney, an essential organ responsible for maintaining proper blood pressure, eliminating metabolic waste, and regulating fluid and electrolyte levels, plays a pivotal role in human health. However, kidney diseases pose a significant global health concern, marked by noteworthy sex-related disparities. These disparities arise from the intricate interplay of genetic and hormonal signals linked to the nuanced biological and physiological differences that shape the diverse landscape of kidney health and disease experiences between men and women.

Notably, disparities extend beyond susceptibility to kidney disease and related risk factors, encompassing various fundamental biological processes integral to kidney functionality. These include aging, cell apoptosis, and the orchestration of key homeostatic systems, such as blood pressure, fluid balance, and the hypothalamic–pituitary–adrenal axis [1–9]. Several factors contribute to these sex-related differences, including the presence or absence of a Y chromosome, variations in gene expression, inheritance patterns of the mitochondrial genome, and differences in neurohormonal activity [10].

Akin to cardiovascular research, the role of sex is frequently overlooked in both preclinical and clinical studies related to kidney health and disease. Historical biases in trials have favoured predominantly male participation, perpetuated by the assumptions that sex-related differences primarily pertain to reproductive biology, and that hormonal fluctuation in females introduces excessive experimental variability. This practice persisted despite an FDA mandate requesting the inclusion of females in clinical trials since 1993 [11]. Nevertheless, a paradigm shift is underway, acknowledging the significance of comprehensively considering sex differences in research settings. Initiatives such as the Committee on Understanding the Biology of Sex and Gender Differences, formed by the Institute of Medicine, USA, in 1999, advocates for expanding the “research on sex at a cellular level” from birth to death and across species in an attempt to eliminate “barriers to advancement of science in health and illness” [12]. Moreover, emerging evidence underscores the existence of sex differences in kidney function and disease, highlighting the imperative to incorporate sex as a significant variable in research [13]. The National Institutes of Health (NIH) has since 2015 mandated the consideration of sex as a biological variable in all NIH-funded research, encompassing its inclusion in study design, analysis, and reporting [14]. Following the NIH call, many journals have revised their policies to include sex-specific reporting [1, 15]. Horizon Europe, as a part of its Gender Equality Strategy 2020–2025, mandates the default integration of sex as a research variable and its evaluation under excellence criteria [16]. Other global granting agencies have also recently followed similar inclusive policies regarding sex-specific representation in research [7].

**Fig. 1:**
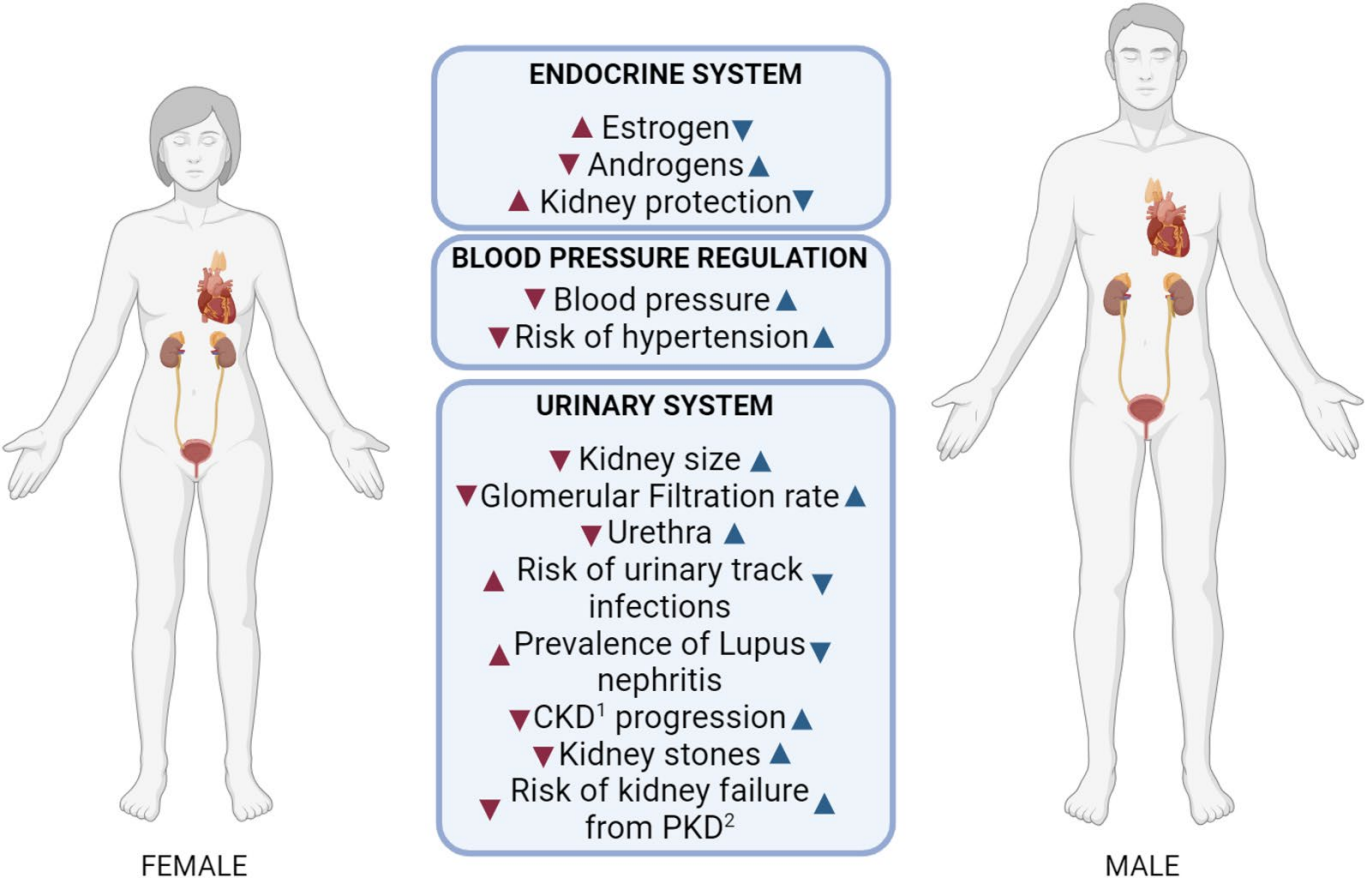
Known differences in kidney physiology, hormonal regulation and prevalence of kidney related disorders between the sexes. ^1^chronic kidney disease, ^2^polycystic kidney disease

Clinical disparities in kidney health and diseases are not solely attributed biological sex, but also include the intricate interplay between biological sex and socially constructed gender (see Sect. "Sexual dimorphism of the kidney"). These disparities manifest in various aspects of kidney disorders, influencing physiology, hormonal regulation, disease prevalence, and even responses to medications that are shown in Fig. 1 [3, 5, 17–22]. Recognizing sex-related differences in drug metabolism and response underscore the necessity to tailor medications and treatment strategies based on a patient's sex.

Investigating the intricate relationship between sex and kidney (patho)physiology holds considerable promise, encompassing both sex-specific hormones and gender-specific stressors, such as perceived stress that have been linked to a lower quality of life in men [23]. Conversely, women were found to employ coping strategies that help maintain a higher quality of life. Moreover, recent evidence suggests that the impact of sex hormones on kidney function may vary depending on the underlying disease and its stage. Therefore, understanding the multifaceted role of sex in kidney disease requires an comprehensive exploration of multiple factors involved, offering insights into disease mechanisms and paving the way for more safer interventions that cater to all individuals and their particularities.

While the machinery responsible for renal transport functions remains relatively underexplored, physiological differences between male and female have been reported (Sect. "Sexual dimorphism of the kidney") and the implications of these differences are explored in Sect. "Clinical implications of sexual dimorphism in kidney function". In vitro kidney models, coupled with advancements in stem cell and organoid biology, tissue engineering, and organ-on-chip technology, offer unique opportunities to investigate sex-based differences in kidney function and diseases (Sect. "Engineered kidney models"). Interrogating sex-specific attributes in these models stands to provide insights into the mechanisms contributing to sexual dimorphisms in humans (Sect. "Design criteria for sex-specific in vitro kidney models"), enhancing our understanding of the role of sex in health and disease.

## Why do we see sexual dimorphism in humans?

To comprehensively address sexual dimorphism, it is crucial to distinguish between sex and gender. Sex pertains to biologically determined characteristics, such as chromosomes, sex organs, and endogeneous hormone profiles, [2, 24], while gender encompasses socially constructed attributes [8, 25]. Importantly, both sex and gender deviate from a strictly binary model, with various sexes, such as Turner syndrome (X), Klinefelter syndrome (XXY) and XYY or XXXY syndromes [26], challenging traditional notions. This review primarily focuses on comparing the two biological sexes, male (XY) and female (XX), referred to as men and women, respectively.

**Fig. 2:**
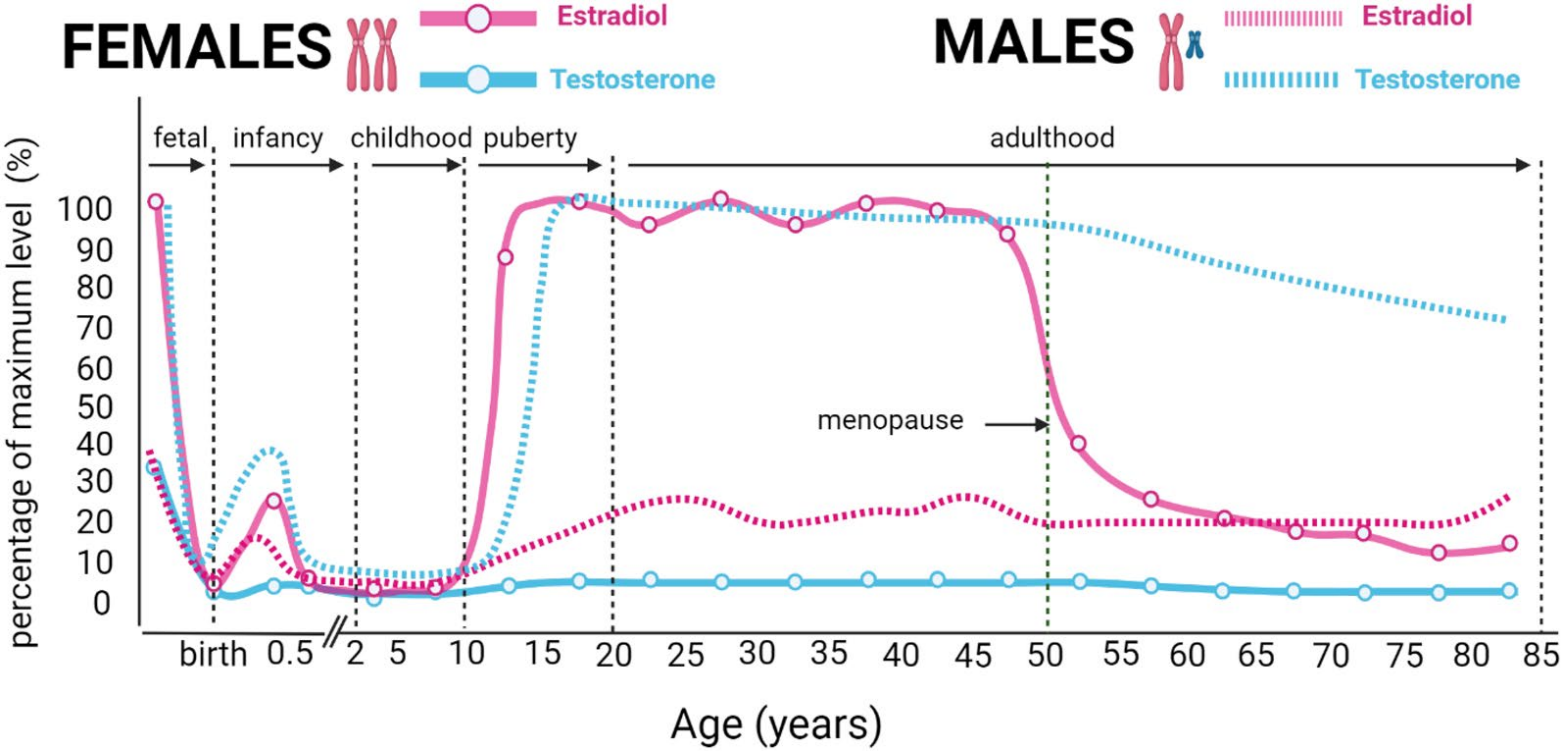
Approximate testosterone and estradiol levels in the plasma of females and males from before birth up to the age of 80. Levels are shown as a percentage of the maximum mean of testosterone and estradiol, respectively. The figure is adapted from [9]

**Fig. 3:**
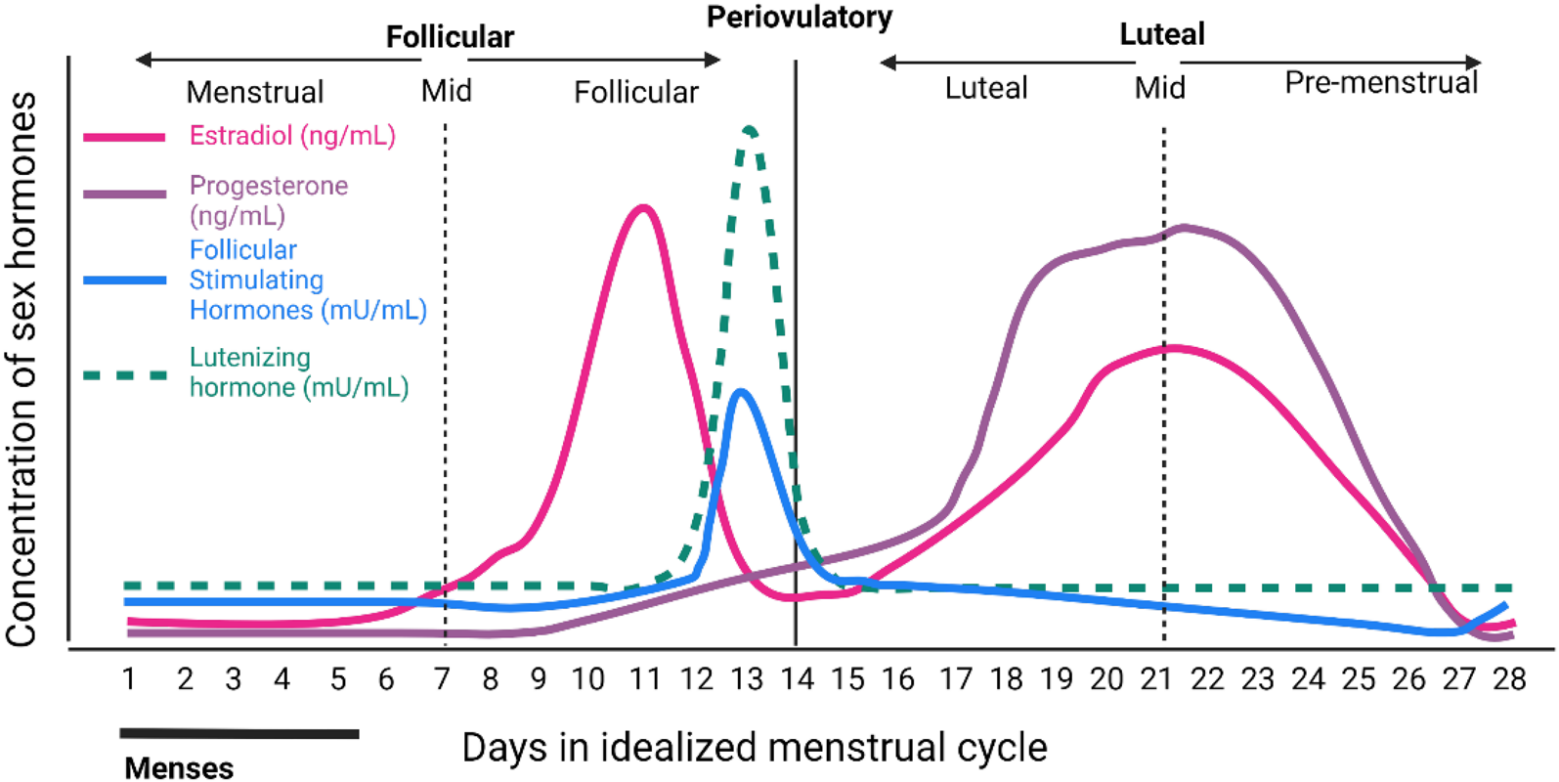
Idealized female hormone cycle of 28 days. Changing levels of estradiol (ng/ml), progesterone (ng/ml), luteinizing hormone (mU/ml), and follicular stimulating hormone (mU/ml) are shown. Adapted from [43]

Sexual dimorphism in humans originated from the allocation of sex chromosomes at conception, where the presence of a Y chromosome leads to the development of male genitalia while the absence of a Y chromosome results in female genitalia. Throughout life, sexual dimorphism is further shaped by differences in gene expression, epigenetics, sex hormone levels, and environmental factors (see Table 1**).** Epigenetic mechanisms, including as X-chromosome inactivation and imprinting, contribute to sex-specific gene expression during fetal development [26]. Sex steroids, such as testosterone and estradiol, significantly influence gene expression via their respective receptors on the cell surface, leading to additional epigenetic changes [8]. Hormone levels undergo fluctuations throughout different life stages, with distinct patterns observed during events such as mini-puberty and puberty, and the decline of hormones around the age of 50 (see Fig. 2**)**. Specifically, in men, the primary sex hormone, testosterone, remains relatively stable around 4.7 ng/ml post-puberty. In contrast, the female hormone system is notably more complex. After puberty, females undergo a 28-day cycle of progesterone, luteinizing hormone (LH), estradiol, and follicle-stimulating hormone (FSH) **(**Fig. 3), with estradiol levels fluctuating between 30 and 800 pg/ml during this cycle [42]. Lifestyle, aging, and pregnancy then further accentuate the differences.

**Table 1:** Contributors to sexual dimorphism in humans with XX and XY sex chromosomes including examples of kidney physiology and pathology

XX	XY
Sex chromosomes [8, 27]:	
• Two X chromosomes • More severe effects in X-linked recessive disorders such as X-linked dominant hypophosphatemic rickets and Fabry disease [27]	• One X and one Y chromosome • SRY gene on Y chromosome regulates testis development
X chromosome inactivation [27–29]: Silencing of one chromosome from gene expression through histone modification and DNA methylation to balance X- linked gene dosage between sexes	
• The inactivated X chromosome is not identical in all cells (mosaicism) • Distribution of X inactivation is unequal or shifts over time (skewing) • Genes escape inactivation. Mutation/alterations may lead to kidney disorders such as Alport Syndrome [27, 29, 30], X-linked nephropathy [27], etc	• Ubiquitous Y gene expression • No need for X chromosome inactivation, but a more severe manifestation of Alport Syndrome, if present
Imprinting [28, 31]: Differential gene expression is dependent on the parent the gene was inherited from. Abnormal imprinting can lead to an increased risk of Wilms tumor and autosomal dominant polycystic kidney disease (sex-independent in rats [32])	
• Both maternal and paternal imprinting of X chromosomes	• Only maternal X chromosome imprinting
Sex steroids[4]: Sex steroid hormones such as estradiol, testosterone, progesterone, luteinizing hormone (LH), and follicular stimulating hormone (FSH) are present in both sexes. Sex steroids influence a variety of signalling pathways, permanently changing cell epigenetics	
• High estradiol levels after puberty until levels drop drastically at menopause. It is generally accepted that estrogen has a protective effect on kidneys [33–35]	• High testosterone peaks at puberty and remains stable with a continuous decline
Lifestyle and aging[6, 8, 36]:	
• Differences in lifestyle accumulate over lifetime as differences in epigenetics, DNA methylation, histone modification, chromatin architecture, and miRNA expression. For example, a high sodium diet could lead to an increased risk of chronic kidney disease [37] • Due to aging, there is loss of functioning nephrons in the kidney. Differences in aging between sexes are recorded but mechanisms poorly understood	
Pregnancy and lactation [8, 38, 39]:	
• Due to the additional workload for the fetus, there are alterations in glomerular filtration rate, electrolyte and balance during pregnancy	
Menstrual cycle [40, 41]:	
• The increase and decrease of estrogen and progesterone levels during the different phases of the menstrual cycle can lead to change in blood flow and fluid balance, retention of more sodium and loss of iron

## Sexual dimorphism of the kidney

Sexual dimorphism in the kidney pertains to the physical and functional differences between male and female kidneys, despite similar kidney anatomy [44]. Notable differences include the relative kidney weight, which is smaller in female than in males, and a lower total nephron count in females [22]. Remarkably in female children, glomeruli, responsible for blood filtration, are significantly larger than in age-matched male children [45]. These variations in filtration rate and size demands for the inclusion of sex-specific cross-validation models used to estimate glomerular filtration rate (GFR), whether creatinine- or cystatin C-based, across all age groups [46, 47]. Here, we explore the primary functional dimorphisms in the kidney.

### Kidney growth

Recent studies have shown that sex hormones affect the developmental phase of kidney and might influence kidney health in the future [48–51]. For example, the kidneys of testosterone-treated female mice histologically show increased weight, due to a thickening of the cortex, primarily caused by hypertrophy in glomeruli and the convoluted tubules [51]. This is further elucidated by a study that associated sexual dimorphism with kidney growth. In nephrectomised rats, male remnant kidneys grew at a much higher rate than their female counterparts [48]. Testosterone was determined as the primary force behind this regrowth. However, in a renal study to track the proliferation in mesangial cells, testosterone showed statistically insignificant effect while estrogen had modest effect on proliferation and also suppressed total collagen synthesis [49].

Growth hormone (GH) plays an influential role in renal growth and physiology [48, 52]. Insulin-like growth factor (IGF-1) is the most studied growth factor synthesised by GH and also independent of GH. GH and IGF-1 knockout animals display abnormalities such as reduced kidney weight and smaller glomeruli in mice [52, 53]. The GH/IGF-1 axis is also important for glomerular filtration and enhances the filtration rate through decreased renal vascular resistance. Estrogen in a known agonist of GH in both men and women and it inhibits the transcription signalling of GH [54]. However in somatostatin knockout mice, Adams et al. reported the enhancement of GH in both male and female mice, therefore suggesting a promoting role of estrogen [55]. In pre-menopausal women taking oral estradiol, reduced IGF-1 concentrations are noted [56]. Puberty leads to an increase in IGF1 which has been associated with increased kidney weight in male rats [57]. Although inconclusive, these studies show the differential effect of sex hormones on kidney growth. Exhaustive reviews on the role of GH-IGF-1 axis on kidney development can be found at [52, 53, 58, 59]. It is thus important to consider the interlinked influence of sex hormones on the GH/IGF-1 axis and kidney growth and development.

### Transporter machinery

Transporters, proteins on the cell membrane facilitating solute uptake, absorption and secretion display sexual dimorphism, influencing urine composition. Veiras et al*.* found that female rat proximal tubuli have higher levels of Na^+^/H^+^ exchanger 3 (NHE3) and lower levels of Na^+^-Pi cotransporter 2, aquaporin-1 (AQP1), and claudin-2 compared to male rats counterparts. In contrast, the distal tubuli of female rats have higher levels of Na^+^/Cl^−^ cotransporter (NCC), claudin-7, and epithelial Na^+^ channel (ENaC) α- and γ-subunits [60]. These transporters are highly specific and efficient, with their coordinated activity being pivotal in determining sex-dependent solute handling. Modelling studies indicate that to induce urine excretions identical to those seen in the male rat nephron model, a variety of transporter activities in the female rat nephron model must be up or regulated in certain segments of the nephron.

**Fig. 4:**
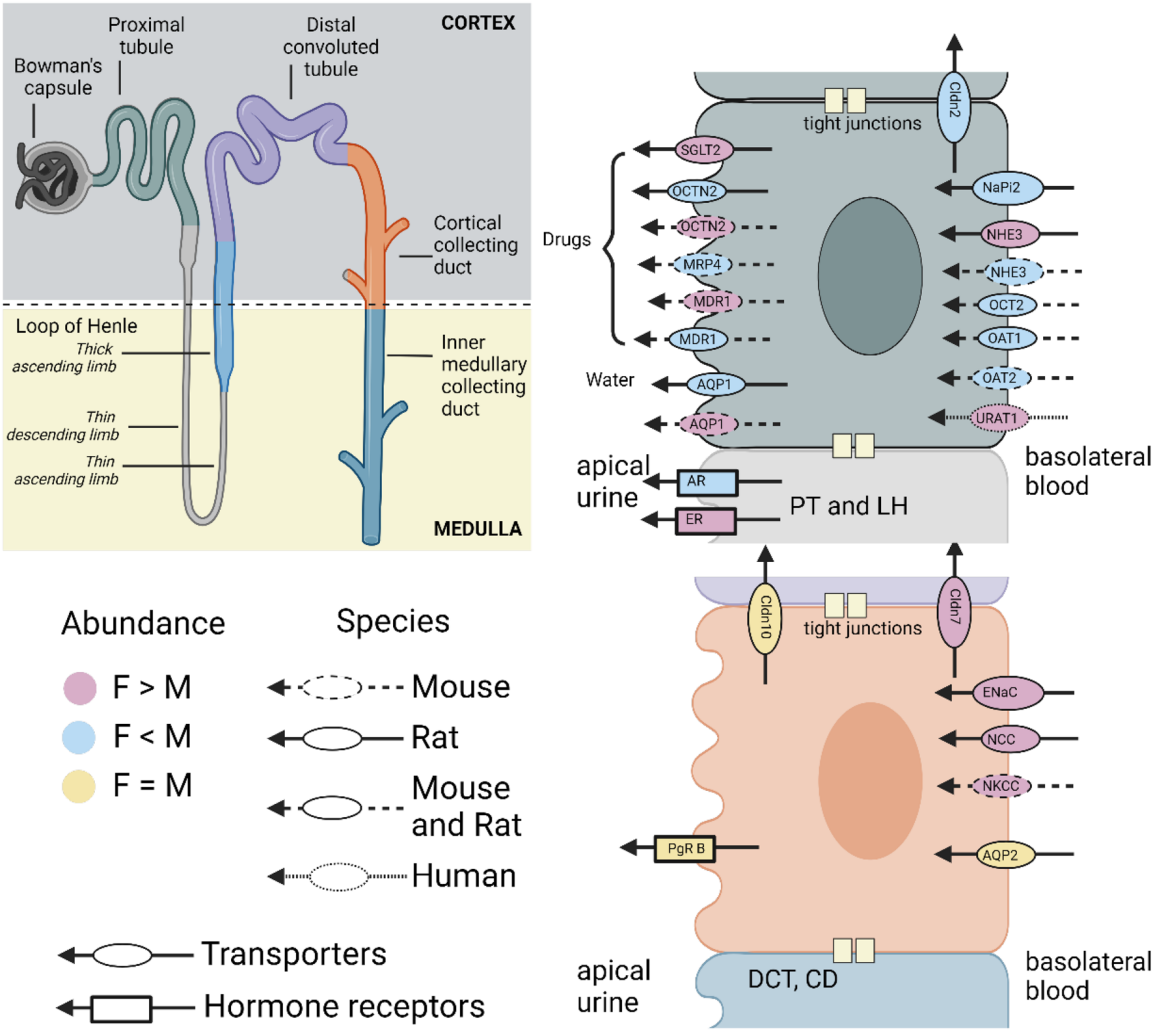
Main transporter and receptor activity in the proximal tubule cells of the nephron, the most important site of drug and endogenous metabolites exchange in the kidney and distal tubule cells. Sex-based differences in activity of these transporters has been reported by sex, species and abundance

The variable transporters activity between sexes lead to different outputs via mathematical modelling. As such, the female proximal tubule reabsorbs fewer solutes and less filtered volume compared to male rats, whereas, a greater proportion of Na + and water transport occurs in the distal tubule and the thick ascending limb, overall leading to a similar urinary output [61]. These sex-specific transporters patterns persist even under a high-sodium diet, with females expected to exhibit 40% lower NHE3 and 200% higher NCC abundance [62]. Hormones like estradiol, progesterone and prolactin stimulate NCC expression, resulting in higher NCC activity in female rats [63]. Similar abundance in NCC activity has also been seen in female mice [64] and could very well be extended to humans. An overview of transporter activity is provided in Table 2**,** with the complexity of (sex-specific) transport mechanisms along the nephron detailed in Fig. 4**.**

**Table 2:** Protein abundance by species and sex

Species	Proteins	Abundance	References
Mouse	SLC17A3, SLC3A1, SLC22A28, SLC1A6 SLC39A5, SLC6A19, SLC7A7, SLC7A8, SLC3A2	M > F F > M	[65, 66]
Rats (pregnant and lactating)	ENaC ↑, Na–K-ATPase ↓	–	[67]
Rats (pregnant)	Urinary angiotensin [1–7] ↑, AQP1 ↑	–	[68]
Rats	MDR1, OAT1, OAT2, OCTN2 SGLT2	M > F F > M	[69]
Mouse	MDR1, OCTN2 OAT2, MRP4	F > M M > F	
Dog	MDR1	F > M

### Renal RAAS system

The renal function under renal RAAS exhibits a sex bias, with males show upregulation of the gene expression of renin, angiotensin-II and aldosterone, leading to more pronounced rise in blood pressure when exposed to angiotensin-II. Conversely, females display enhanced expression of the counter-regulatory arm of the renal RAAS system, including angiotensin-covering enzyme (ACE) 2/Ang(1–7)/MasR and AT2R, which may contribute to the lower prevalence of hypertension and cardiovascular disease in pre-menopausal women compared to men [70, 71].

Estrogens and androgens levels play a significant role in the control of blood pressure through the RAAS system [13]. Estrogen increases angiotensinogen levels, while negatively affecting renin, ACE, AT1 receptor, and aldosterone. It also inhibits the activation of NAD(P)H by ACE2, reducing the production of reactive oxygen species and helping maintain vascular function. On the other hand, progesterone competes with aldosterone for mineralocorticoid receptors, potentially reducing salt and water retention and lowering blood pressure. Testosterone, in turn, raises plasma levels of renin and ACE, increasing the risk of hypertension in males.

Pre-menopausal females experience protection against high blood pressure, renal, and cardiovascular diseases, protection that diminishes [13, 70] due to the decrease in estrogen levels, leading to increased vascular tone, inflammation, and oxidative stress. Therefore, estrogen replacement therapy may be beneficial for post-menopausal female suffering of hypertension or cardiovascular disease, although potential risks associated with hormone therapy, such as increased risks of ovarian and breast cancer, thromboembolic disease, need to be considered [72].

Cardiac and renal dysfunction occur mostly simultaneously, where pathophysiological changes in one can promote injury in the other [73, 74]. Unsurprisingly, there are sex differences noted in the cardiac physiology across life [75] and these differences are cascading into sex differences in the pathology of cardiovascular diseases [76, 77]. We can expect the renal pathology to also be affected by such cardiovascular sex-differences and these need to be studied in a “cardiorenal unit” to model the cardiorenal syndrome [74, 78].

### Sex hormone receptors in the kidney

Sex hormone receptors in the kidney play a crucial role in regulating hormone activity, influencing signalling cascades. Sex steroids, like testosterone, estradiol, and progesterone affect the expression of renal transporters. For instance, testosterone has been shown to affect the expression of NHE3, NKCC2, AQP2 [79], estrogen and androgen stimulate calcium membrane transport in tubule cells in vitro [80], while progesterone competes with aldosterone for mineralocorticoid receptor, affecting electrolyte transport in the kidney [81].

Localizing receptors for sex hormones in the mammalian kidney has been complicated by the limited abundance of the receptor proteins and/or their mRNAs, as well as the complex relationship between hormone receptors and specific actions within the kidney, which remains a subject of ongoing research. An overview of the localization of estrogen, androgen, and progesterone receptors in the kidney, the sex of the cells they were detected in, and any recorded sex-specific abundance differences is presented in Table 3 [82].

**Table 3:** Sex hormone receptors in the human kidney

Sex	Species	Receptor type	Localization	F vs M	References
M	Human	ERα, ERβ	Interstitial cells, collecting duct	–	[82]
F & M		ERβ	Cortex > Medulla, Convoluted PT	–	
F & M		PgR B	Medulla > Cortex, Glomerulus, distal tubule, interstitium (nuclei)	F = M	
F & M		AR	–	F < M	
F & M		AR	Distal tubule	–	
F &M		AR	–	F < M	[83, 84]
F & M		AR	–	F = M	[83]
F & M	Rats	ERα	–	F < M	[85]
F & M	Mouse	AR	–	F = M	[86, 87]

Receptor type, localization within the kidney, and relative distribution between males and females are marked. Male sex is denoted as M, female sex as F. Estrogen receptors alpha and beta, progesterone receptors, and androgen receptors are denoted as ER, PgR, and AR respectively

Alterations in hormone receptor activity can have significant consequences, as disruptions to the normal functioning of the kidney can lead to a range of clinical conditions. For example, changes in mineralocorticoid receptor activity can result in disorders such as hypertension and electrolyte imbalances. Additionally, modifications in the expression of renal transporters can lead to impaired kidney function, contributing to the development of chronic kidney disease (CKD) and other related comorbidities.

### Response to inflammation

Sex differences in kidney diseases are significantly influenced by genetic and hormonal factors, impacting inflammatory and immune responses. Estrogens generally protect against kidney damage by reducing pro-inflammatory cytokines like TNF-α and IL-6, and increasing anti-inflammatory cytokines such as IL-10. Conversely, androgens exacerbate inflammation, leading to more severe kidney damage in males. Studies show that estrogen treatment mitigates renal injury in female rats, while testosterone aggravates renal inflammation and fibrosis in male rats [88–93]. A particular study reveals that in men under 60, after adjusting for conventional CKD risk factors, a higher neutrophil to lymphocyte ratio was independently associated with a higher risk of CKD, with an adjusted odds ratio of 1.48 [94]. This association, however, was not observed in women or in older individuals of either sex, indicating a sex- and age-specific inflammatory response in CKD susceptibility. Sex differences are also evident in the renal T cell profile, where hypertensive males exhibit more pro-inflammatory T cells compared to females, who possess more regulatory T cells associated with lower blood pressure and protein excretion [95]. Angiotensin-II increased pro-inflammatory cytokines in male kidneys only. These differences are influenced by blood pressure management, suggesting that hypertension may drive sex-specific immune responses in the kidneys. Further information on the role of sex-specific inflammatory response in kidney diseases can be found in [50, 96, 97]. Altogether, this underscores the contrasting roles of sex hormones in kidney response to inflammation, with estrogens providing protective effects against renal damage and sclerosis.

## Clinical implications of sexual dimorphism in kidney function

### Implications for nephrotoxicity screens

Nephrotoxicity, the toxic side effects of chemicals on the kidneys, can have significant clinical consequences such as disruption of regulatory mechanisms, like glomerular filtration rate or the RAAS system [98]. The loss of nephrons, the functional units of the kidneys, can lead to impaired kidney function, and in severe cases, kidney failure. The kidney is particularly vulnerable to drug-induced toxicity because of its high metabolic activity, the concentrations of solutes in its tubuli and its drug clearance performance [99].

As discussed earlier, sex differences in drug transporters expression can results in differences in drug clearance rates and nephrotoxicity. For example, females have a 25% lower systemic clearance of the anticoagulant lepirudin than male [100] and are more susceptible to nephrotoxicity induced by the antibiotic amikacin [101]. Peri-menopausal females are at significantly higher risk for cisplatin-induced nephrotoxicity than males, a phenomenon that is explained by the higher estrogen level [18].

### Implications for kidney diseases

Sex-based differences manifest significantly in various kidney-related diseases, with CKD being a noteworthy example. Affecting over 10% of the global population [102], CKD poses a substantial morbidity and mortality burden due to its contribution to end-stage kidney disease (ESKD) and cardiovascular diseases [103]. CKD exhibits sex-related differences in pathophysiology, symptoms, complications, and therapeutic efficacy. While females have a higher incidence of CKD, males tend to progress more rapidly towards ESKD. The influence of estrogen on nitric oxide metabolism, imparting renoprotective properties, is suggested as a potential explanation for this difference. Estrogen is believed to play a major role in the slower progression of CKD in females, with a noted decline post-menopause [3, 93].

Research indicates that testosterone may play a differential role in kidney health across sexes. Women with hyperandrogenism as observed in polycystic ovarian syndrome (PCOS) [104] display an increased risk of developing CKD [105]. In diabetes, women are more prone to develop renal complications, albeit an approximately 10-year delayed onset to males. Sex hormones and sex-specific gene expression are thought to be the contributing factors [106]. For instance, olfactory receptor, Olfr1393, displays a stronger phenotype in female rats, altering the glucose/glutamine metabolism, and thus, potentially explaining sex differences in kidney disease in diabetes [20]. Other studies that identified genes overexpressed or underexpressed in the individuals with renal diseases are summarized in Table 4. This suggests that testosterone's impact on kidney function and the development of CKD might be mediated by different mechanisms in men and women.

**Table 4:** Genes associated with various kidney diseases that show sex dependence

Gene	Associated disease	References
JAG1, HES1	Diabetic nephropathy (human)	[113]
GAP43, FOXD1, POSTN, EMP2, **PTGIS**, MPPED2, **AKR1C2**, **PTHLH**, ANKRD6, LRRC17, **VIM**	Chronic kidney disease (human)	[114]
CA9, DGCR5, EGLN3, SLC16A3, SLC5A3, SPAG4, VEGF, SEMA5B, PFKP, PRAME, MUF1, MGC45419, GPR54, FABP7	Clear cell renal cell carcinoma (human)	[115, 116]
Tpbg, RFP, HLA-DQA1, SCNN1B, SLC15A2	X-linked Alport syndrome (Canine)	[30]
VEGFa, LRPAP1, AGTR1, SLC12A1, CUBN, DAO1, KCNJ1, CASR, PTHR1, SLC12A3, FXYD2, KLK6, SCNN1A, SCNN1B, ATP6V1B1, AVPR2, AQP2	Polycystic kidney disease (mouse)	[117]

Genes related to pregnancy (specific to females) and spermatogenesis (specific to males) are highlighted in bold

Genetic studies further support this notion. For instance, genetically predicted testosterone levels are associated with an increased risk of CKD in men, but not in women [107]. This differential effect might be influenced by the interaction between testosterone levels and other factors like sex hormone-binding globulin. Additionally, testosterone therapy in clinical settings has been observed to influence kidney-related biomarkers such as calprotectin and phosphate, potentially reducing inflammation and cardiovascular risks, particularly in men [108].

Animal studies have provided insight into the role of sex hormones in kidney pathology. For example, intact male animals have been shown to develop glomerular injury and proteinuria, whereas females and castrated males are generally protected from such kidney damage [109, 110]. This suggests that androgens might increase susceptibility to kidney injuries, whereas the absence of these hormones due to castration appears to mitigate such risks. This is in agreement with the study by Rogers et al*.* on ovariectomised rats. The study shows reduced angiotensin type 1 receptor binding with increased estradiol in both castrated male and ovariectomised female rats, resulting in reduced angiotensin-II signalling which decreases the susceptibility to vascular and renal diseases [111, 112].

Polycystic kidney disease demonstrated a worse prognosis in men compared to women, partially attributed to the influence of testosterone on cAMP generation, thus stimulating fluid elimination and solute transport [118]. Similarly, although historically men were at higher risk of developing kidney stones, the prevalence has been rising over the last decade, especially for women [5].

X-linked chromosome inactivation in females can lead to Alport syndrome, X-linked dominant hypophosphatemic rickets, X-linked nephropathy and Fabry disease [27, 29]. Female children showed more prevalence of lupus nephritis than their male counterparts, however, the disease tends to be more aggressive in male adults [119, 120]. Pre-eclampsia, in particular, is associated with increased sodium reabsorption, potentially leading to hypertension [121].

### Implications for hormone therapy

Among the adult population worldwide, approximately 0.3–0.6% or about 25 million individuals identify as transgender [122]. Gender-affirming hormone therapy (GAHT) can significantly impact drug dosage profiles and renal function, necessitating careful consideration in clinical settings. GAHT influences serum creatinine levels, a key marker used to assess kidney function and drug clearance. In transgender women receiving feminizing hormones, creatinine levels typically decrease, which can reduce the apparent renal clearance of drugs, potentially increasing their toxicity [123]. Conversely, in transgender men on masculinizing hormones, creatinine levels usually rise, enhancing renal drug clearance and possibly requiring higher dosages to achieve therapeutic effects. In a recent cohort study of transgender and gender-diverse individuals undergoing GAHT, it was observed that feminizing hormone treatments were linked to a 0.069 mg/L reduction in cystatin C levels [124]. This reduction correlates with an increase in the eGFR by 7 ml/min/1.73m^2^, as calculated using the gender-neutral Full-Age-Spectrum equation. Conversely, masculinizing hormone treatments were linked to 0.052 mg/L increase in cystatin C levels, which corresponds to a decrease in eGFR by 6 ml/min/1.73m^2^ [124]. Additionally, estrogen-based GAHT is linked to the development of microalbuminuria [125]. This substantiates that there is a variation in solute absorption in the kidney when exogenous hormones are administered. Animal models of gender transition support the notion of GAHT affecting kidney structure and function. Indeed, cross-sex testosterone therapy in female rats increased glomerular area, systemic blood pressure, and sodium excretion, whereas urine osmolarity and GFR were decreased [126]. In male rats treated with cross-sex estrogen/progestogen therapy, plasma concentrations of urea and creatinine were increased [127].

Thus, GAHT impacts kidney structure and alters serum creatinine levels, which can affect the accuracy of kidney function tests. Given these nuances, different dosing strategies based on renal clearance estimation are recommended for patients undergoing varying durations of GAHT [128]. Accurate interpretation of these changes is crucial for effective and safe medication management in transgender and gender-diverse individuals. Of note, the impact of GAHT on kidney structure and function might contribute to the increased prevalence of kidney diseases in the transgender population [129].

## Engineered kidney models

The development of accurate kidney models is of paramount importance for advancing our understanding of kidney function and disease, as well as for testing the safety and efficacy of new therapies. As mentioned in Sect. "Sexual dimorphism of the kidney", significant sex differences exist in kidney physiology, kidney models must enable the recapitulation of both male and female kidney phenotypes. Animal models, genetically engineered rodents, and in vitro models, such as organoids and cell lines, provide opportunities to investigate sex-specific contribution to kidney function and disease. However, we should note that animal models are needed as clinical data is uncontrolled, has unmatched variety (due to genetics, lifestyle and environmental factors), it is ethically challenging, and it is not easily available.

### Cell sources

Sex differences in kidney function and injury response change with age [17, 131] and the interplay of these factors in in vitro experiments remains unclear. Age- and sex-specific changes in DNA methylation indicate that these factors likely cannot be considered separately [132].

In vitro kidney modelling utilizes primary cells, immortalized cell lines, and induced pluripotent stem cells (iPSCs). Primary cells closely mimic in vivo physiology but are limited by the time and labour-intensive isolation process, and restricted culture due to dedifferentiation and senescence. Immortalized cell lines have been developed to overcome these limitations but often exhibit dedifferentiation resulting in reduced expression of functional markers, such as transporter expression loss, resulting in loss in predictive power. It should be remarked that in human kidney disease research, non-human donor species are more frequent than human cells [133, 134] (see Table 5).

**Table 5:**
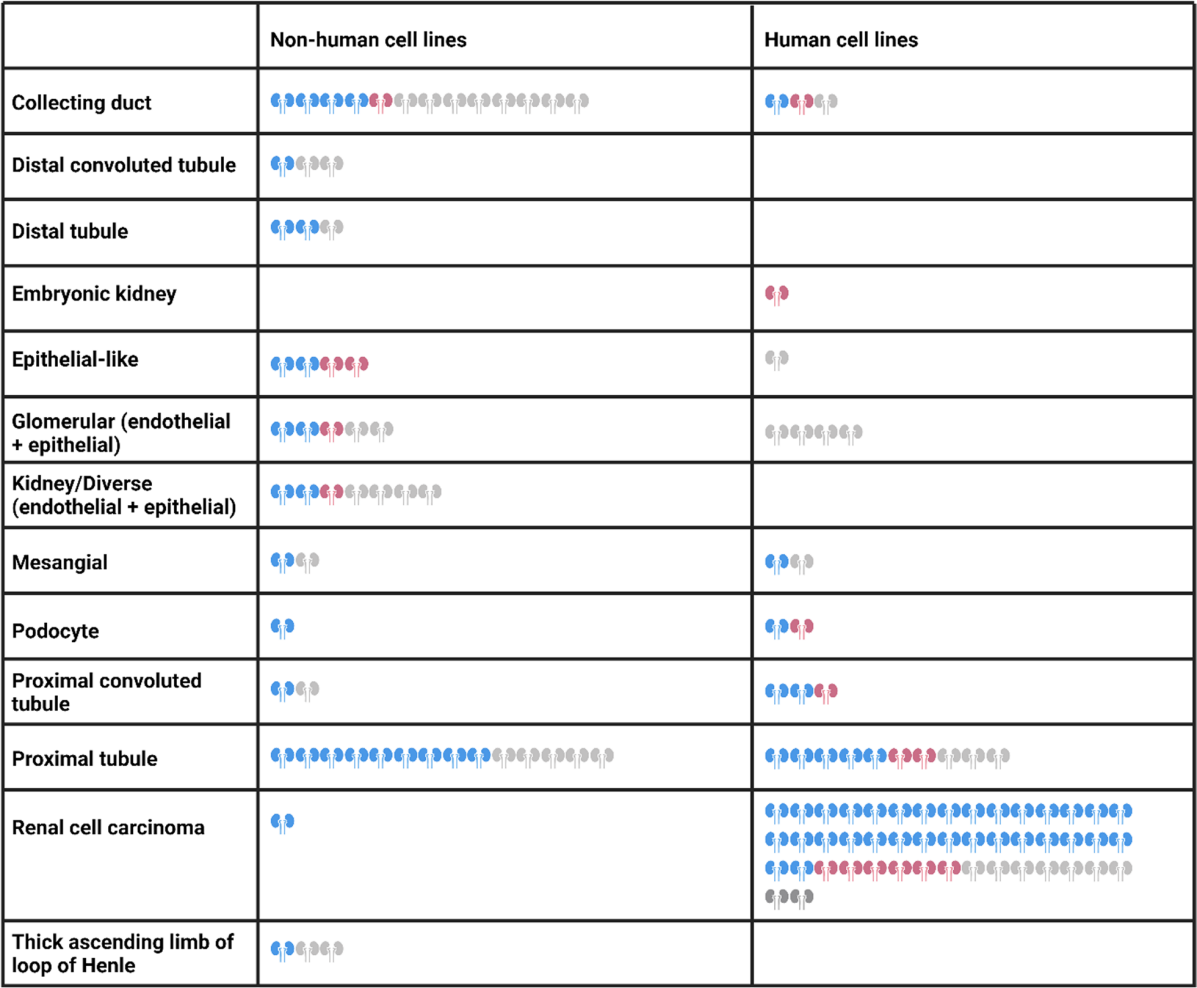
Original nephron segment/cell type and sex of recorded cell lines in Appendix A

One kidney pair represents one cell line. Male cell lines are indicated in blue and female cell lines in pink. Cell lines with no determined sex are indicated in grey

**Fig. 5:**
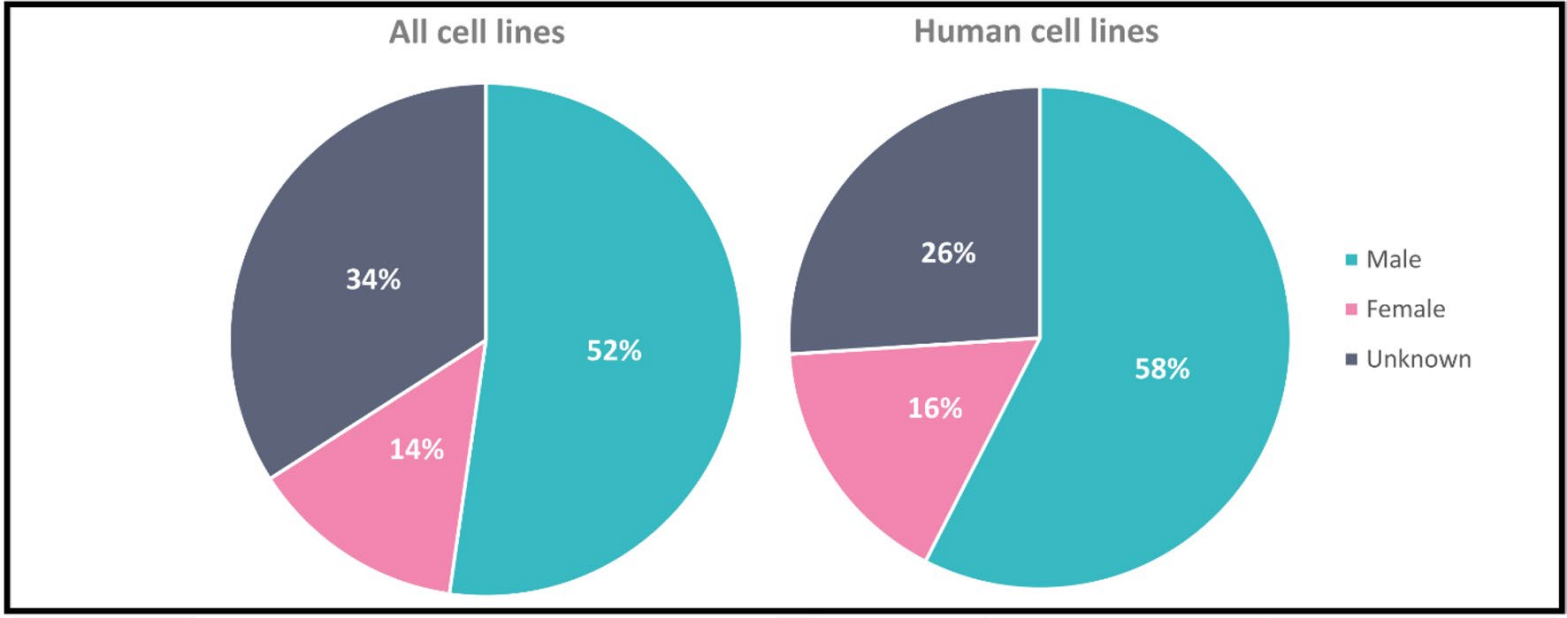
Recorded sex of 131 kidney cell lines for all species (left) found in the cell line database Cellosaurus[130] and specifically 72 human cell lines (right). Cell lines are separated by recorded sex, male or female. Cells for which no sex is recorded are denoted as unknown. Detailed information can be found in the appendix

The sex of the donor is not always disclosed in primary sources for cell lines due to patient confidentiality and ethical guidelines. Instead, for established cell lines, the sex of the donor is typically noted in initial studies, but information may be lost in subsequent publications. Kouthouridis et al*.* found that up to 50% of in vitro studies do not specify the sex of their cells and only 20% use cells of both sexes [42]. To illustrate both the distribution of male and female sex and the number of cell lines with unknown sex, we had a closer look at the *Cellosaurus* database [130]. We identified an unequal distribution of sexes within established cell lines, with about a third of recorded cell lines lacking information about their sex (Fig. 5 + Appendix A). For a quarter of the human kidney cell lines, the sex could be determined, and two-thirds of the remaining cell lines were male.

Sex distribution varied by nephron segment (Table 5) with the sex of all human glomerular cell lines remaining unknown. For the thick ascending loop of Henle, there are neither human cell lines nor cell lines with known female sex. Only one embryonic kidney cell line was noted to be female, no male comparison could be found in the database.

Immortalized cell lines develop chromosomal instability after repeated passages. For several cell lines a loss of the Y chromosome has been recorded, inhibiting their use in sex-specific studies [135].

In additions to primary cells and immortalized cell lines, induced pluripotent stem cells offer a third alternative. Using iPSC technology, various kidney cell types and kidney stem cells can be created from patient-derived material. Cells are isolated and reprogrammed back to pluripotency through exposure to a cocktail of finely tuned factors. Subsequently, these iPSCs can be redifferentiated towards most cell types. Protocols for differentiating iPSCs into various kidney cell types are detailed in Ribeiro et al*.*[136]*.* However, the use of iPSCs is not without limitations. In human pluripotent cells, the expression of the sex-determining Y-chromosome-linked gene SRY in male cell leads to differential autosomal gene expression compared to female cells. This differential expression could potentially affect steroid metabolism and differentiation [137]. Furthermore, the sex of the donors influences the nature of the imprinting defects in iPSCs and their response to culture conditions [138]. We should note that the creation of iPSC-derived kidney cells is time-consuming and resource-intensive [136, 139]. As with primary cells, the availability of knowledge about iPSC donor sex depends on patient confidentiality and ethics guidelines.

### 2D Models

2D cell culture are still the most commonly used model for drug screening and nephrotoxicity studies [140] due to their simplicity and practicality. However, they have limitations in terms of physiological relevance [133, 141]. These limitations encompass the absence of apical-basal polarization and expression of key uptake and efflux transporters [98, 133], as well as lack of cell-ECM interactions which can affect cell proliferation, polarization, migration, signal transduction, and gene expression (142). Additionally, the proximal tubule is often the focus of nephrotoxicity testing. A systematic review by Irvine et al*.* found that of the included in vitro nephrotoxicity studies, 82% studied proximal tubule cells [140]. Although they are particularly susceptible, this fails to capture the variety of nephrotoxicity, which can also affect all parts of the kidney [99]. The development of in vitro kidney models which capture the simplicity, practicality, and speed of 2D models, while delivering translatable results, has therefore long been strived for.

### 3D models

Due to the complexity of the kidney and the presence of different cell types, a multitude of complex kidney models have been recently developed.

#### Organoids

Organoids represent 3D culture system in which stem cells self-organize in vitro to form structural and phenotypical patterns highly similar or identical to their native organs [134, 139, 143, 144]. Kidney organoids can be generated both from iPSCs and adult stem cells (AdSCs). IPSC-derived organoids follow developmental steps to form kidney-like structures, although maturation still remains an issue.

On the other side, AdSCs are established by guiding iPSCs through the developmental steps of the wanted cell population. These organoids, originating from adult tissue, can replicate various maturation features, with a particular enrichment of the distal part (see work on tubuloids [144, 145]), although they do not encompass all segments of the kidney. AdSC organoids are established through the isolation of the cells from the adult tissue, they are maintained for organoid culture through the presence of the niche factors specific to this stem cell type [139, 144, 146]. AdSC organoids are therefore restricted to tissues with maintained stem cell niches [145]. Organoids offer a practical, genetically stable model system for studying kidney development, disease and drug safety, as well as potential regenerative therapies.

First attempts have been made to use kidney organoids in nephrotoxicity studies. Although organoids with multiple different kidney cell types have been developed, markers are missing which are sensitive enough to distinguish toxicity in the different cell types. Organoids are also difficult to perfuse, limiting compound administration [134, 142].

Although not yet reported for kidneys, organoids have been used to study the impact of sexual dimorphisms in other organs. Kelava et al*.* studied the impact of sex chromosomes and sex steroids on neurogenic potential by creating brain organoids from stem cells of both sexes and the addition of androgens and estradiol to the culture medium [147]. Devall et al. compared male and female organoids in a study on the influence of calcium in the context of colorectal cancer [148], while Fitzgerald et al*.* developed endometrial organoids that were responsive to reproductive hormones [149]. These studies emphasize the future impact of organoids as a platform for the study of sexual dimorphism and its impact on potential therapies.

#### Kidney-on-a-chip

Organs-on-a-chip are microfluidic culture devices designed for precise cell culture. They provide continuous perfusion, real time metabolite sensing capabilities and allow mechanical stimulation. The ease of customization and variety of commercial designs, allow for fit-for-purpose strategies. In general, these chips contain perfusable channels separated by an interface (*e.g.* membrane, hydrogel, etc.). Each channel can be seeded with single or multiple cell types to recapitulate physiology accurately, including cell interactions, pressure gradients, flow dynamic, and metabolic and transport functions [150].

Kidney-on-a-chip have been developed for various nephron segments, such as glomerulus, distal tubule, and collecting duct, with a keen focus on proximal tubule. [151]. Proximal tubule models closely reflect renal physiology including solute transport and toxic injury response. [150, 151]. Although, well-characterized on-chip models of the thick ascending limb, interstitial, and cortical collecting duct are yet to be developed, the inter renal segment connection would allow the modelling of the entire nephron, a system that could be more relevant than the sum of its parts. But ongoing work in the co-culture of epithelial and endothelial cells as well as advancements in creating gradients chip platforms have paved the way for more complex and relevant on-chip models [150]. Combining patient-derived AdSC organoids and microfluidic systems enabled molecular and cellular analysis in a personalized disease modelling and drug screening approach [145]. In the meantime, glomerulus models have been created by layering human podocytes and glomerular endothelial cells, exhibiting glomerular functions, such as permeability and selectivity, and long-term culture maintenance [152].

Kidney-on-chip have been offered as an optimal platform for drug nephrotoxicity testing, combining accessibility with physiological relevance [150, 153]. For example, vascularized kidney spheroids with integrated sensors rapidly elucidated nephrotoxicity mechanisms of Cyclosporine and Cisplatin [154].

Organ-on-chip systems are highly customizable and regulatable, they offer unique opportunities to incorporate and study sex differences. A review of organ-on-chip studies by Nawroth et al*.* found that organ-on-chip models are uniquely qualified to study the effect of soluble factors such as hormones, as levels can be varied continuously to mimic the physiological concentration within a tissue [155]. Although a variety of male and female reproductive organs have been modelled in organ-on-chip systems, consideration of sex as a biological variable for other organs is lacking [155, 156]. The review by Nawroth et al*.* found that only 3% of the considered organ-on-chip platforms recorded the use of solely female cells within one model system [155]. No studies were found that used the distinctive benefits of organ-on-chip platforms, such as physical stimulation, sensors, and channel geometry, to study sexual dimorphism. Therefore, the potential of organ-on-chip technology in exploring sex differences in kidneys and nephrotoxicity testing has yet to be fully realized.

#### De-& recellularized kidneys

Kidney ECM plays a vital role in tissue integrity, supporting kidney cells and contributing to kidney. For instance, the glomerular basement membrane, a mixture of collagen and laminin, encompassing the glomerular filtration barrier, supports the activity podocytes, endothelial cells, and mesangial cells [157]. Corresponding to its importance, some attempts at engineering kidney models have focused on preserving and reseeding the ECM of cadaver kidneys. Although there is limited information regarding ECM differences between the male and female in kidney tissues, studies on other organs like bone, brain, heart indicates that ECM composition in health and disease is sexually dimorphic [158–161]. In the future, de- and recellularization of healthy and diseased (male and female) ECM could aid in the functional recapitulation of the specific tissue phenotypes in the laboratory.

#### Biofabricated kidneys

Bioprinting, a technology that combines additive manufacturing methods with biocompatible materials and cells, enables the intricate construction of biological structures. In the realm of kidney bioprinting, a range of bioinks and biomaterial inks have been developed, including hydrogels composed of decellularized ECM. Various bioprinting techniques have been employed for creating kidney models, with extrusion, microfluidic and droplet-based printers being the primary choice [162]. A particularly intriguing approach involves the use of coaxial nozzles and sacrificial ink, enabling the formation of hollow tubes with distinct inner and outer layers for the recreation of nephron structures [163].

Bioprinting is often used in combination with other technologies to produce geometrically complex models. For instance, the integration of microfluidic and bioprinting techniques has led to the development of intricate, perfusable convoluted renal proximal tubule with customizable geometries [164]. Extrusion bioprinting has also been utilized to create kidney organoids in a highly uniform and high-throughput manner, making them suitable for nephrotoxicity testing [165]. Organovo Inc., for example, has designed a 3D-printed tissue specifically for nephrotoxicity testing, comprising an interstitial layer, a basement membrane, and a polarized layer of renal epithelial cells staked atop of each other. This setup offers insight into cell–cell interactions during nephrotoxicity-related events [166]. While studies on sex-specific biofabrication of kidney have not been published to date, the advantage of bioprinting, such as its ability to create highly customizable shapes in a reproducible, high-throughput manner, are expected to play a crucial role in future research.

### In silico models

Next to 2D and 3D models, significant progress has been made in creating in silico renal models. These mathematical models encompass the kidney at the organ level and its functional unit, the nephron, offering insights into the roles of renal transporters [167, 168]. For example, Weinstein et al*.* developed spatial mathematical models for tubular transport, allowing calculations of cytosolic concentrations, transport fluxes, and epithelial permeabilities [168–171]. In silico models have been employed for predicting nephrotoxicity, albeit in a generalized manner, without specific information on the type of adverse effects or toxic dosage [172, 173]. Hallow et al. developed a whole kidney compartmental model to assess the time response of single nephron GFR, sodium and glucose balance, tubuloglomerular feedback under various conditions [174–176]. Mathematical models of protein uptake along the proximal tubule like the one described in Edwards et al*.* have contributed to our understanding of proteinuria [177]. Moreover, Nordsletten et al*.* proposed a 3D rendering of kidney vasculature based on CT scans rat kidney [178]. Pannabecker et al*.* took an innovative approach by combining the vascular architecture of the inner medulla with an epithelial transport model of Layton et al., to explain the urine concentrating mechanism [179]. While the majority of current in silico models focus on rat kidneys due to the availability of data, they generally lack the incorporation of sex differences, primarily to the absence of detailed experimental data. Notably, the Layton group’s mathematical models have provided insights into sex differences in rat kidneys, including transporter pattern and their functional implications, circadian rhythm regulation, and hypertension [19, 61, 180, 181]. Their in silico model exploring the effect of pregnancy on female rat kidneys revealed that renal transporter sex differences might prepare the kidney for the metabolic demands of the pregnancy [182]. These models underscore the importance of considering sex in biomedical research and the necessary for sex-specific experimental data to calibrate and validate the in silico models. Hence, pharmacokinetic, safety, efficacy, and toxicity data acquired from a non-human test model, might drastically misrepresent sex-based mechanisms in humans, so caution must be exercised when extrapolating such data to the sex/species not included in the experiment [69].

## Design criteria for sex-specific in vitro kidney models

Creating kidney models that account for sex-specific differences offers numerous advantages for understanding sex-related disparities in renal (patho)physiology. In vitro models provide a highly controlled environment where the influence of genetic, epigenetic, and hormonal differences between sexes can be thoroughly examined. This approach can enhance the safety screen for clinical drug testing for female participants, as women, especially pregnant ones, are more likely to experience adverse drug reactions. Improved preclinical safety and efficacy testing can also save significant costs in drug development, as up to 19% of drugs fail during phase III clinical trials due to previously undetected nephrotoxicity issues [140, 183].

The use of iPSC allows for patient-specificity, ensuring a virtually endless supply of patient-specific cells that can be expanded and directed into various cell types, including renal lineages. It also enables focused investigation on genetic, epigenetic, hormonal variables by circumventing environmental and behavioral confounding factors common in clinical and preclinical studies. These models based on human biology, bypass species-specific effects often encountered in animal studies. Moreover, these models facilitate precise manipulations of experimental variables, including factors like the timing and dosage of hormone (pre)conditioning, biomaterials properties, and gene regulation, leading to detailed explorations of sex-specific distinctions in renal physiology in vitro. This approach enhances our understanding of kidney health and disease within the context of sex, providing a more comprehensive perspective [94, 139].

### Integrating sex as a biological variable of cells lines

**Fig. 6:**
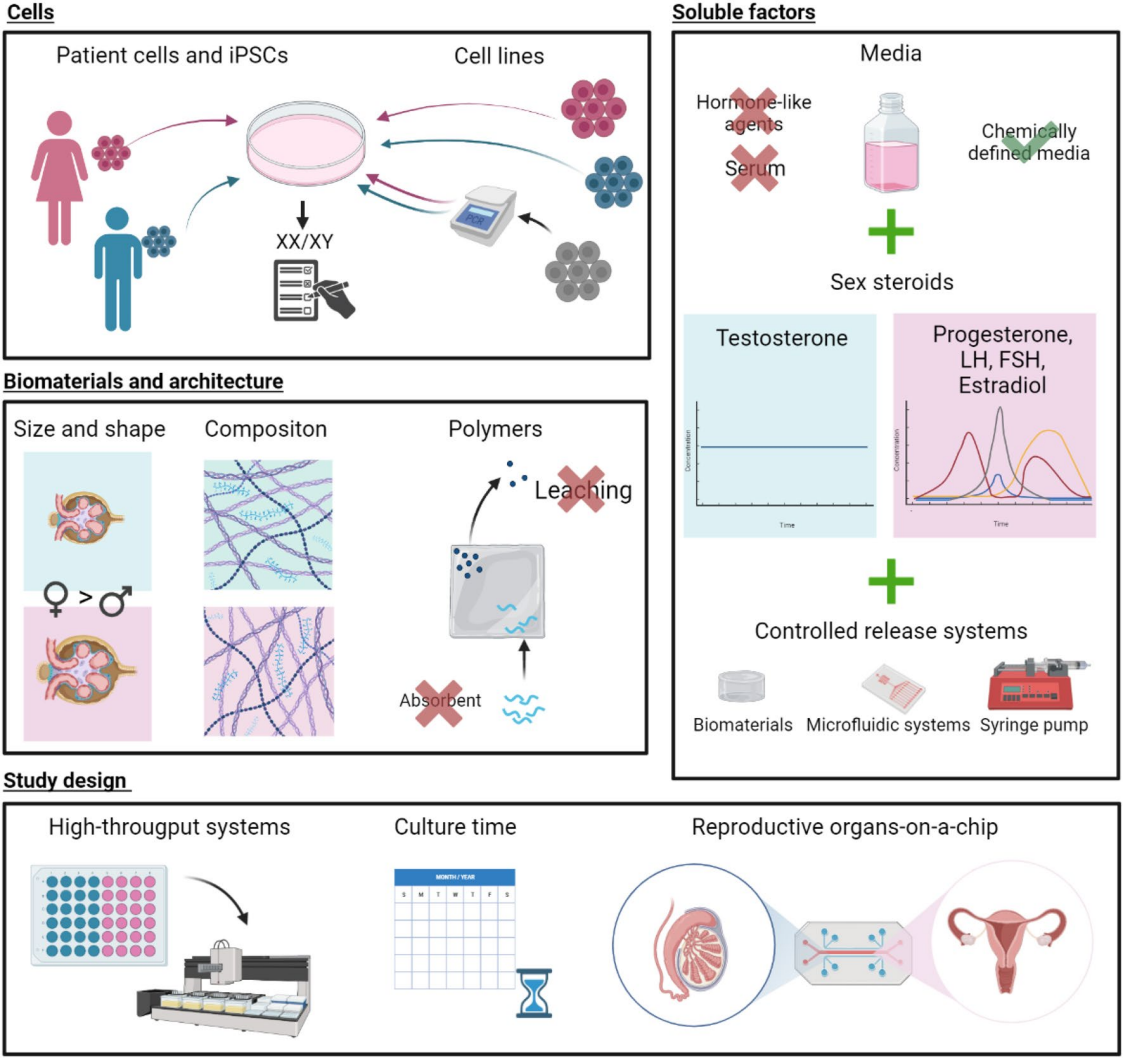
Opportunities to incorporate sex differences in vitro models through cells, soluble factors, biomaterials, and architecture as well as study design. Male sex is indicated with blue, female sex with pink. Undetermined sex is indicated in grey

The choice of cells in creating in vitro models is often the first step when considering incorporation of sex as a biological parameter. Recording the sex of the donor of the cells lines is crucial. If sex information is not provided by vendors, employing karyotyping and XY/XX mRNA analysis (e.g. SRY for male and XIST for female) could be considered. It is advisable for journals to mandate the reporting of sex when using cell lines [184]. Whenever possible, cells of both sexes should be incorporated into the study, with sex of the cells becoming a biological variable (Fig. 6).

### Incorporating sex hormones in culture media

Incorporating sex hormones into culture media presents a valuable opportunity to introduce sexual dimorphism into model systems. Levels of sex steroids fluctuate throughout different stages of life, and in females, throughout the menstrual cycle, pregnancy and lactation as illustrated in Fig. 3. [42], warranting an increased attention to female-biology specific models. To accurately replicate hormone levels in tissue, rather than serum levels, specific tissue levels should be evaluated to prevent overdosing [185]. Advanced controlled drug delivery methods can be adapted for the precise delivery of sex hormones in in vivo models. Microfluidic devices offer enhanced control over hormone levels [186, 187].

The inclusion of hormone-producing gonads in human-on-chip models is promising for replicating complex hormonal dynamics. Promising examples are a female reproductive tract-on-chip model which replicated the 28-day menstrual cycle [188] and the multi-organ chip co-culture of liver and testis cells [156]. While adding sex-specific hormones to replicate hormonal changes, it is essential to eliminate other hormones or hormone-like agents that may confound the experiment. Standard media often contain sex hormones, and their concentrations can vary between batches [189, 190]. Removing hormone-like agents is also crucial to prevent experimental variability. Developing serum-free media formulations is challenging but offers consistency and eliminates batch variability. Careful consideration should be given to all medium additions to minimize hormone activity and the potential impact of hormone-like agents on study outcomes. [191]. Kim and Audet developed a serum-free media formulation for hematopoietic cells using a high-dimensional search algorithm. This shows one way the development of serum-free media alternatives could be optimized [192, 193]. Another benefit is that the use of serum-free media has been associated with decreased inter-donor variability [192]. Using chemically defined media also removes medium batches as a variable between experiments.

Unwanted sex hormone-like agents in media also need to be considered. Sex hormone receptors are present on kidney cells, as elaborated in Table 3, and a variety of non-hormone compounds can bind and activate those receptors [194]. Phenol red, included in most standard media as a pH indicator, is a known estrogenic compound. It has been shown to affect cell proliferation rates, stem cell differentiation, and the efficacy of some anti-cancer drugs. Male and female cells respond differently to exposure to phenol-red. To further complicate matters, concentrations of phenol red differ between media types and suppliers [42, 191]. To exclude the effect of hormone-like agents on study outcome, all medium additions should be carefully considered for potential hormone activity (Fig. 6).

### Biomaterials selection

Sexual dimorphism also needs to be considered when choosing the biological materials often obtained from animal or human sources. ECM composition differences between males and females have been shown for several tissues, such as the brain and heart [159, 161]. Such information about the kidney is lacking and this knowledge gap should be closed. Nevertheless, it is essential for correct modelling to reconstruct potential differences in vitro. In the realm of synthetic polymers, the mechanical and chemical characteristics can be tailored to meet specific requirements. When using natural polymers, the sex of the source should be recorded and if necessary, the sex of the tissue used to obtain ECMs libraries and cellular model should be matched [184].

When designing 3D micro-physiological models, the sex differences in kidney architecture can also be considered. Glomerular size is sex-dependent in both human children and mice [45, 195]. 3D printing offers the opportunity to incorporate such differences within models. It is critical that materials used for microfluidic devices are biocompatible, inert, and non-leaching. When using microfluidic devices in combination with carefully controlled hormone levels, it is important to consider all involved materials (Fig. 6). Polydimethylsiloxane (PDMS), a frequently used polymer for microfluidic devices, absorbs hydrophobic molecules and may thus change the concentration of added hormones. For the construction of biochips, materials that exhibit less absorption of hydrophobic compounds, like polycarbonate and poly(methyl methacrylate) should be favoured [150]. The materials used in cell culture could also be a source of hormone-like agents. The polystyrene used for most single-use cell culture plastics has been shown to release weak estrogen and may weaken the effects of added estrogen [191].

### Study design

The study design is severely affected by the inclusion of sex as a biological variable. The simple inclusion of models for both sexes doubles the sample size. Should sex be considered a variable and tested for, the sample size could multiply by six [42]. This highlights the importance of high throughput systems for the incorporation of sex as a biological variable. Shaughnessey et al. have developed a high throughput system to evaluate kidney toxicity, exemplifying ways in which multiple independent samples can be assessed simultaneously [196].

The long female cycle additionally complicates the study setup and brings up several questions. Because hormone levels can affect physiology, the days of the female cycle most optimal for testing should be explored. It is also not yet known how long cells must be conditioned within a hormone cycle to fully adapt to it. If this period is too long, the integration of the female cycle into cell culture experiments could be too complex for many setups. Lastly, going through the entire cycle would prolong in vitro studies dramatically. This makes it worthwhile to include experiments in a study design to explore hormone concentrations and configurations of hormones, which allow for an approximation of the menstrual cycle effect.

As discussed in Sect. "Sexual dimorphism of the kidney", kidney function changes with age in a sex-dependent manner, including drug-dependent sensitivity [17]. For high-fidelity kidney models of all potential patients, different stages of human life and the according sex-specific kidney function may need to be represented (Fig. 6).

First steps have been taken to create sex-specific toxicity models. In the case of Baert et al. a co-culture of liver and testis organoids was set up to test for the reproductive toxicity of a drug metabolite from the liver. A design focusing on sex-specific engineered cardiac model was proposed by Lock et al. [26]. This highlights both the importance of incorporating multi-organ systems and sex-specificity in toxicity tests [156].

## Discussion

Sex-specificity needs to be included in design of kidney models, in vitro and in vivo. The need for sex-inclusive studies in kidney research is empashized by the difference in renal transporter expression, presence of sex hormone receptors and their effect of sex hormones on kidney function, as seen in the RAAS, sex-specific response to nephrotoxicity and kidney disease progression. Therefore, the development of human sex-specific models for drug testing and regenerative studies is of utmost importance. Better in vitro and in silico model systems will enable the study of individual contributing factors.

Novel techniques such as organoids, kidney-on-a-chip, biofabrication, and recellularization offer excellent opportunities for sex-specific modelling. However, these opportunities remain underutilized, and most studies using these models do not account for sex-specific differences. The potential value of sex-inclusive studies of the kidney has been exemplified by Clotet-Freixas et al. and Sandhu et al. [20, 118]. Because these studies recapitulated the sex differences in kidney disease, they were able to give insights into previously unknown disease mechanisms in diabetes and polycystic kidney disease.

Despite the potential value of sex-inclusive studies of the kidney, there are major challenges ahead in incorporating sexual dimorphism in engineered kidney models. For example, the availability of kidney cell lines specific to each part of the nephron for each sex is severely limited. A comprehensive library of cell lines with known sex and a wide demographic range would enable easier modelling. Another challenge is the culture method. In particular, truly sex-neutral culture methods need to be created (as a first step as well as control). They should be equally conducive to the culture of male and female cells, and the medium and serum used should include no hormones or hormone-like agents. As shown in Sect. "Sex hormone receptors in the kidney", sex hormones also show differential effects in males and females, constituting an additional challenge. Leaching or absorbent materials should be avoided. To such a culture method, sex-specific signals like hormones can then be added. Down the line, controlled release systems will enable the incorporation of more advanced hormone fluctuations to mimic e.g. the menstrual cycle. Importantly, it is not yet clear to what degree physiological circumstances need to be imitated to recreate sex-specificity. While the use of cells of both sexes and sex hormones might be enough in some cases, a more intricate setup might be needed for others. A deeper understanding of the origin of sexual dimorphism and their evolutionary purpose in the kidney could provide the needed insight.

The development of advanced, sex-specific in silico models also faces significant hurdles. One of the major challenges is the lack of available data on sex-specific differences in renal physiology and disease. Comprehensive studies exploring transporter differences, the quantity and location of sex hormone receptors, and ECM composition and structure in the male and female human kidney are missing. The current data heavily relies on animal models, but the interspecies variation calls the applicability of such data into question. As the quality of in silico models relies heavily on the data of previous studies, better experimental data from human models will also help the development of accurate human in silico models of the kidney.

While this review focused on the kidney, most of the opportunities for sex-specificity in vitro are equally applicable to other tissues of the body. As the importance of sex differences in health and disease becomes more well-known, the methods to include them in in vitro models will become more common and refined. This will enable a better understanding of the underlying mechanisms of disease throughout human physiology, overall leading to more effective treatments that account for sex-specific differences.

## Perspectives and significance

Incorporating sex as a variable when assessing pharmacodynamics and pharmacokinetics in drug development is crucial for ensuring dosing accuracy across diverse populations. Because the kidney plays a key role in clearing drugs from the body, understanding its function in both males and females is essential. As pharmaceutical research begins at the laboratory bench, it is vital to consider potential sex differences when designing in vitro experiments. In addition, it is important to report sex-disaggregated outcomes, even when sex disparities are not found, to improve the fundamental understanding of drug responses and potential side effects. Moving beyond the bench to pre-clinical and clinical testing, there is a need for rigorous research into sex-specific pharmacokinetics and pharmacodynamics, in healthy and pathological settings, including in particular women in their pre-, peri- and post-menopausal period, pregnancy as well as individuals receiving gender-affirming hormone therapy. In summary, incorporating sex as a variable from bench to bedside would enhance the overall safety and (patient-specific) efficacy of drugs, ultimately benefiting patients worldwide.

## Conclusions

In conclusion, the inclusion of sex-specific differences in engineered in vitro and in silico kidney models is vital for advancing our understanding of kidney physiology and disease. It is also essential for developing more accurate and predictive drug development and safety assays. To this end, a framework has been proposed for creating sex-specific kidney models (in vitro and in silico), which includes cell selection and characterization, the surrounding extracellular environment (structure and hormones) and appropriate study design.

## Data Availability

Not applicable.

## Appendix A

Renal cell lines in the database Cellosaurus[130]. Cell lines are sorted alphabetically first by donor species, then by name. Parent cell lines are listed in bold, and popular cell line children are noted when applicable. Information about donor sex, species, and cell or tissue origin is added whenever available. Cells lines with known use in nephrotoxicity testing are highlighted in light grey.

## Abbreviations

AKR1C2: Aldo–keto reductase family 1 member C2
ANKRD6: Ankyrin repeat domain-containing protein 6
AGTR1: Angiotensin-II Receptor Type 1
AQP2: Aquaporin 2
ATP6V1B1: ATPase H + Transporting V1 Subunit B1
BAD: Bcl-2-associated agonist of cell death
CASR: Calcium Sensing Receptor
CUBN: Cubilin
DAO1: D-Amino Acid Oxidase 1
DAXX: Death domain-associated protein
DGCR5: DiGeorge syndrome critical region gene 5
EGLN3: Egl-9 family hypoxia-inducible factor 3
EMP2: Epithelial membrane protein 2
FADD: Fas-associated protein with death domain
FOXD1: Forkhead box D1
FXYD2: FXYD Domain-Containing Ion Transport Regulator 2
GAP43: Growth-associated protein 43
GPR54: G protein-coupled receptor 54
HES1: Hairy and enhancer of split 1
HIG2: Hypoxia-inducible protein 2
HOXC10: Homeobox protein Hox-C10
HLA-DQA1: Major histocompatibility complex, class II, DQ alpha 1
JAG1: Notch ligand jagged 1
KCNJ1: Potassium Inwardly Rectifying Channel Subfamily J Member 1
KLK6: Kallikrein-Related Peptidase 6
LRPAP1: Low Density Lipoprotein Receptor-Related Protein Associated Protein 1
LRRC17: Leucine-rich repeat-containing protein 17
MGC45419: Uncharacterized protein C14orf166
MPPED2: Metallophosphoesterase domain-containing protein 2
MUF1: Mitochondrial ubiquitin fold modifier 1
Olfr1393: Olfactory receptor family 139 subfamily 3 member 93
PFKP: Phosphofructokinase platelet
PITX1: Paired-like homeodomain transcription factor 1
POSTN: Periostin
PRAME: Preferentially expressed antigen in melanoma
PTGIS: Prostaglandin I2 (prostacyclin) synthase
PTHLH: Parathyroid hormone-like hormone
P53: Tumor protein p53
SCNN1A: Sodium Channel Epithelial 1 Alpha Subunit
SCNN1B: Sodium channel, non-voltage-gated 1 beta subunit
SEMA5B: Semaphorin 5B
SLC12A1: Solute Carrier Family 12 Member 1
SLC12A3: Solute Carrier Family 12 Member 3
SLC15A2: Solute carrier family 15 member 2
SLC16A3: Solute carrier family 16 member 3
SLC5A3: Solute carrier family 5 member 3
Tpbg: Trophoblast glycoprotein
VEGF: Vascular endothelial growth factor A
VIM: Vimentin
